# Death from Chloroform: Fatty Degeneration of the Heart

**Published:** 1870-01

**Authors:** J. F. Miner


ART. II.—*Death from Chloroform: Fatty Degeneration of the Heart.* By J. F. MINER, M. D.

The following case of apparent death from chloroform is of value, in a statistical point of view at least, and has some features of interest connected with the general questions of, to whom, and under what circumstances, is it safe and proper to administer chloroform.

Oct. 20—E. B., aged about forty, received injury in coupling cars, the right hand being crushed between the car bumpers. He was brought into my office, and upon examination was told what was before quite apparent to him, that amputation of the fore-arm would be necessary.

He requested chloroform to be given, and I commenced its administration, while my private students arranged for the operation. The chloroform was given by dropping it upon a napkin and holding it at sufficient distance to allow ample atmospheric air. After breathing it, for a few minutes, he became talkative and finally considerable excited, requiring restraint. He soon had a condition of rigidity of the muscular system, drawing back the head, as in partial convulsion. This condition attracted my attention and caused me to withdraw the chloroform, though he had the second before, talked loudly and profanely, and did not appear enough under its influence for me to think of permanently discontinuing it. His appearance was now peculiar and cannot be described by words. I noticed that there was something in his respiration and general condition which I had never before observed in patients inhaling the vapor of chloroform. I had merely time to say to my assistants his pulse is very weak, when I was obliged to finish my sentence by saying, it has stopped. Respiration ceased after one or two short inspiratory efforts, and my patient was dead.

Such is a brief history of what occurred before *post mortem* examination, in which I was assisted by a number of my professional friends.

Prof. T. F. Rochester, at my request, has kindly furnished the following *post mortem* appearances, and the results also of a careful microscopic examination of the tissues of the heart:

*Autopsy.*—Six hours after death. Body of a well formed man, slightly muscular, about forty years of age. Weight, estimated at one hundred and forty-five pounds. Surface cold. Cadaveric rigidity slight. Hæmatic suggilation very marked. Hand and lower forearm terribly crushed, lacerated and denuded.

*Sectio Cadaveris—Thorax.*—Lungs collapsed, no adhesions, crepitant, pallid—healthy. Pericardium contained about one ounce of straw colored serum. Two white spots (maculæ lacteæ) on cardiac reflexion of right ventricle, the larger, an inch in diameter. *Heart.* Right auricle and right ventricle enormously distended with dark and fluid blood. Fatty deposit about base of heart, a little more than common. Ventricles *red,* but doughy and inelastic, and feeling like tallow when punctured with a tenaculum. *Heart opened.* A large quantity of dark fluid blood escaped from both auricle and ventricle of right side; left auricle and ventricle *empty.* The tricuspid valve and the aortic and pulmonic valves were normal. The mitral valve was functionally adequate, but was the seat of a slight abnormal deposit, probably fibrous. Weight of heart, emptied, ten and a half ounces. The walls of the auricles and ventricles were about normal in thickness, but the belly of the left ventricle slightly exceeded half an inch. The cavities were somewhat more capacious than usual. The muscular fibre looked pretty well, but was soft and *mashed* under the finger like tallow.

*Microscopic Examination.*—Portions of the right and left ventricles and of the columnæ carnæ of the left ventricle were inspected: The muscular fibre was very distinct, but looked like a wax cast of the same. It was very thickly interspersed with fat granules, and oil globules were very abundant in the field. The liver was healthy; the kidneys normal, but engorged with dark fluid blood.

*Remarks.*—The extreme fluidity of the blood, and the fact that both the right auricle and right ventricle were enormously distended, is to say the least, peculiar—and affords much room for speculation. There was fatty muscular degeneration to an advanced degree, and yet it is very doubtful if this could have been detected by careful physical exploration, conducted at any time before the occurrence of the sad accident.

In most of the deaths by chloroform, where *post mortem* examinations have been made, "fatty degeneration of the heart" is reported. This condition must be much more frequent than is generally supposed; and where there have been no signs or symptoms to indicate its existence, it cannot be discovered by manipulation or auscultation."

Fatty degeneration of the heart, without other change, is a pathological condition, which usually gives during life no signs adequate to its discovery. If we could know that such disease was present, we might refuse chloroform and ether, and choose the suffering incident to operation rather than the risk of anæsthesia. But a well marked case of fatty degeneration of the heart, has dangers which even anæsthesia might lessen. All surgeons are familiar with the case reported by DeSault, who lost a patient about to be lithotomized. He was supposed to have died from sheer fright, when this distinguished surgeon only marked the line of intended incision with the thumb nail. If this patient had been inhaling anæsthetic vapor, there would have been no doubt, that *it* was the agent which produced the fatal result.

In the case reported, the fright and excitement which the injury and anticipated operation caused, was adequate to produce instant death, in a case of fatty degeneration of the heart, or in any other condition of this organ where its strength or function was greatly interfered with. It is a common occurrence to see patients with structural disease of the heart drop down and die suddenly from the slightest causes of excitement and increased circulation. It may be that the increased heart action, caused by excitement and fear, is as dangerous in such cases as the anæsthetic. Formerly we used to hear of *fear* and mental *shock* proving instantly fatal, but no mention is made in those cases of the condition of the central organ of the circulation. The histories of persons being sentenced to be bled to death, who actually died on hearing the water trickle into the basin, which they supposed to be blood issuing from their veins, after their arms had been slightly pricked, although no vessel had been opened, are familiar to all. If we had given our patient no anæsthetic, and the result had been the same, we should have been reasonably well satisfied with attributing the death to mental shock, or mental shock joined with the shock of injury, and if not thus satisfied, we should have regarded the explanation as complete when the condition of fatty degeneration of the heart was taken into consideration. It *seemed* at first a case of unmistakable death from chloroform, and the *post mortem* examination was commenced with the expectation that no organic change would be found to explain the result. The history of death from chloroform would seem to leave no doubt of its having proved fatal in a considerable number of cases, where no fault in administration or disease of patient could be urged in explanation or extenuation. It is perhaps as desirable to make careful discrimination, and exclude from our list of deaths from chloroform all cases which do not rightfully belong to it, as it is to trace to it all deaths which it causes. We want to know the amount of risk involved in the inhalation of chloroform in conditions of perfect health, and what effect it has also, in conditions of disease. But few of the cases of death from chloroform have been subject to careful *post mortem* examination, and consequently their histories may be said to be in great degree imperfect.

